# Supplementary material for: Inhibition of Drp1/Fis1 interaction slows progression of amyotrophic lateral sclerosis
Source: EMBO Mol Med. 2018 Jan 15;10(3):e8166. doi: 10.15252/emmm.201708166 (PMC5840540; doi:10.15252/emmm.201708166)
Supplement: Supplementary file 1 — Appendix [file EMMM-10-e8166-s001.pdf]

Appendix

Inhibition of Drp1/Fis1 interaction slows progression of Amyotrophic lateral sclerosis

Authors

Amit U. Joshi[1], Nay L. Saw[2], Hannes Vogel[3], Anna. D. Cunnigham[1], Mehrdad Shamloo[2], Daria Mochly-Rosen[1]

Affiliation

[1]Department of Chemical and Systems Biology, Stanford University School of Medicine, Stanford, CA 94305

[2]Behavioral and Functional Neuroscience Laboratory, Department of Neurosurgery, Stanford University School of Medicine, Stanford, CA 94305

[3]Department of Pathology, Stanford University School of Medicine, Stanford, CA 94305

Contact information:

mochly@stanford.edu

Running title (50 characters including spaces): Drp1/ Fis1 interaction in ALS models

Table of Contents:

Appendix Figure S1

Appendix Figure legend

Appendix Table 1

Appendix Figure S1

A Histology

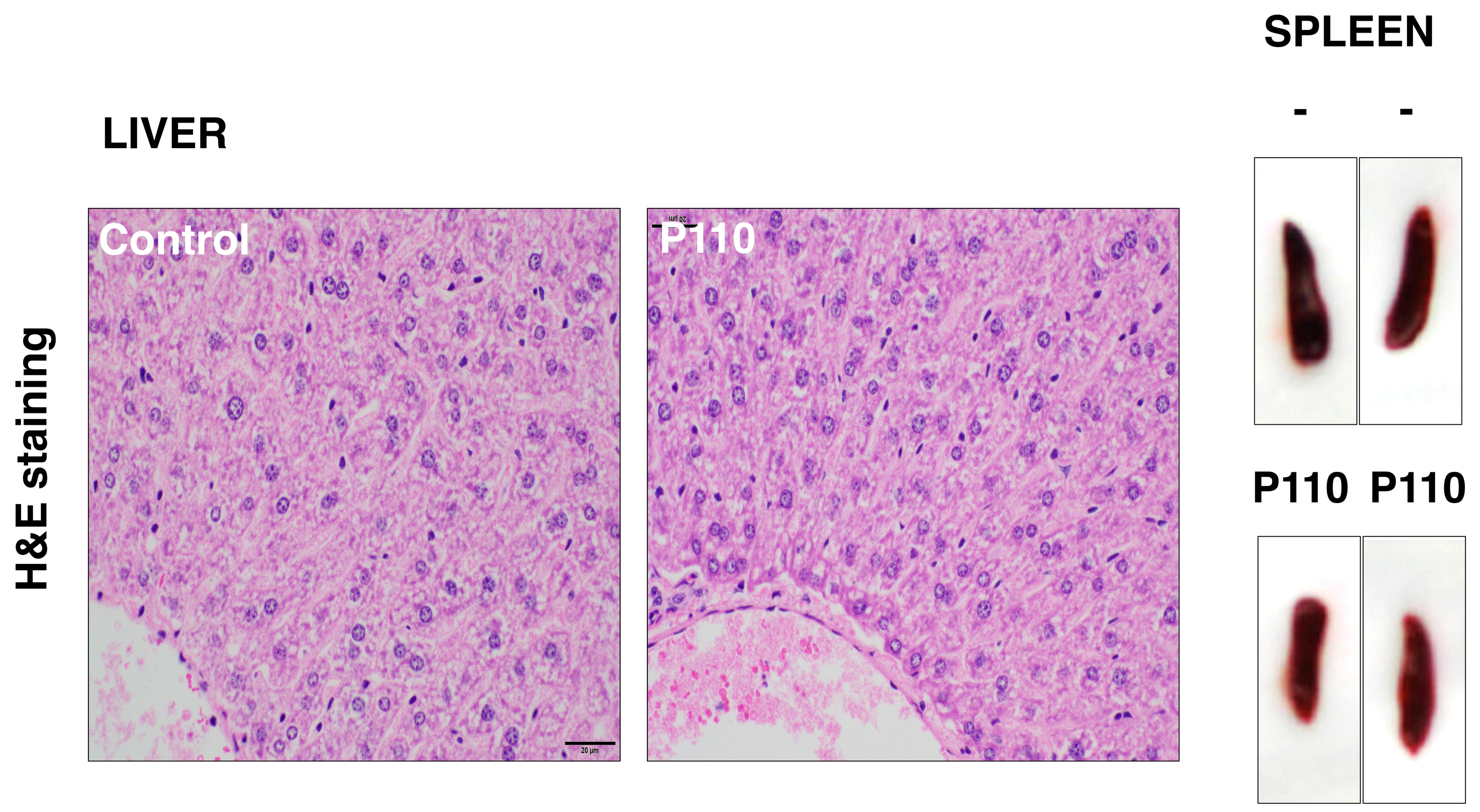

D Mitochondrial fission and fusion proteins

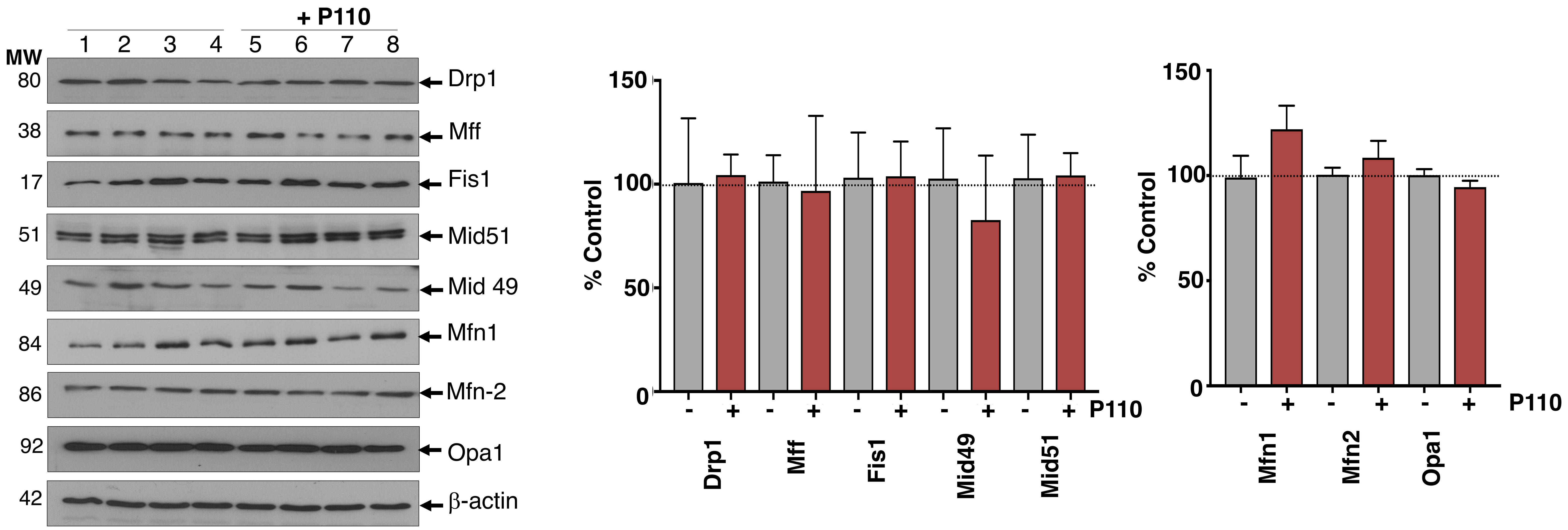

B Mouse Activity Chamber

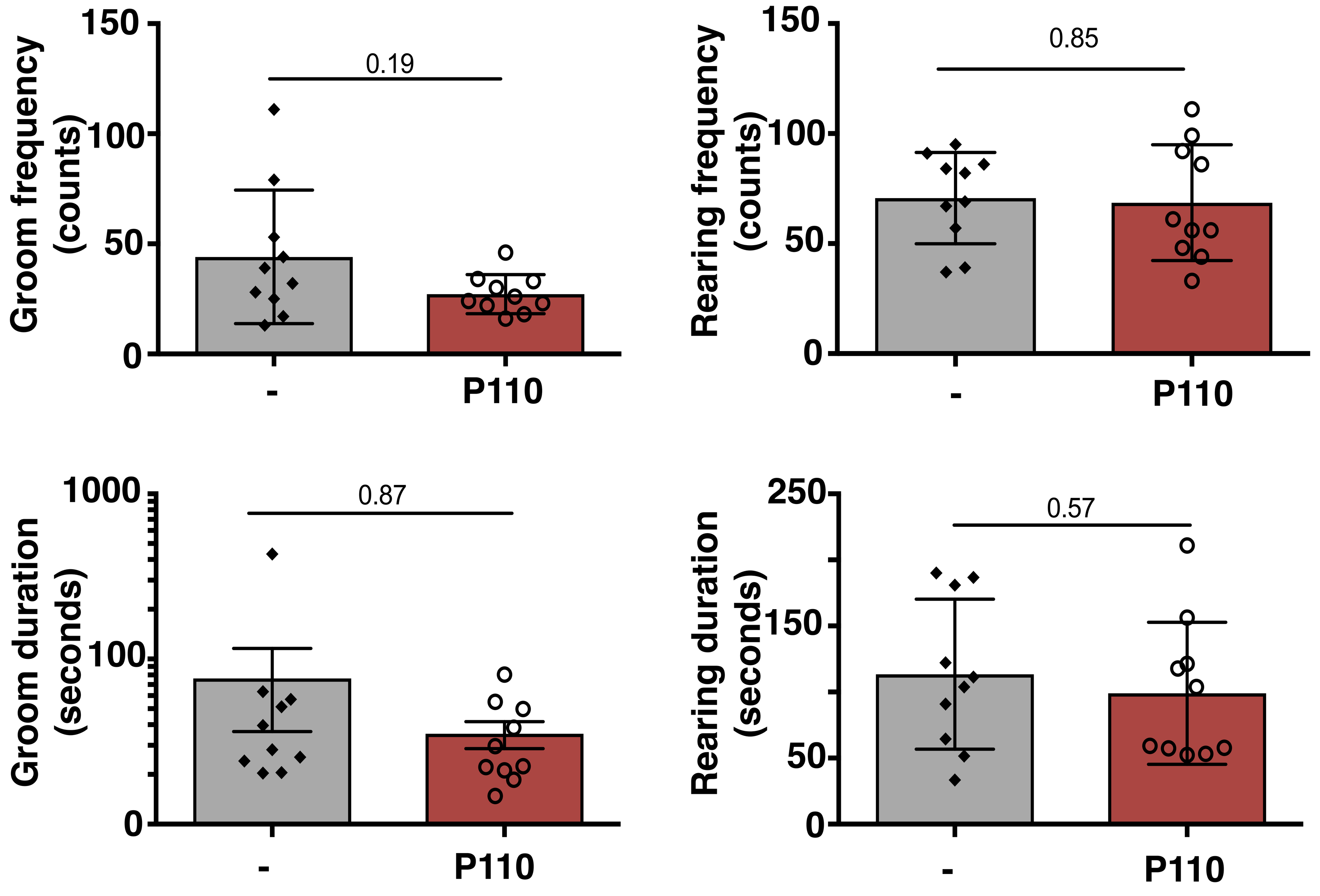

E Mitochondrial health markers

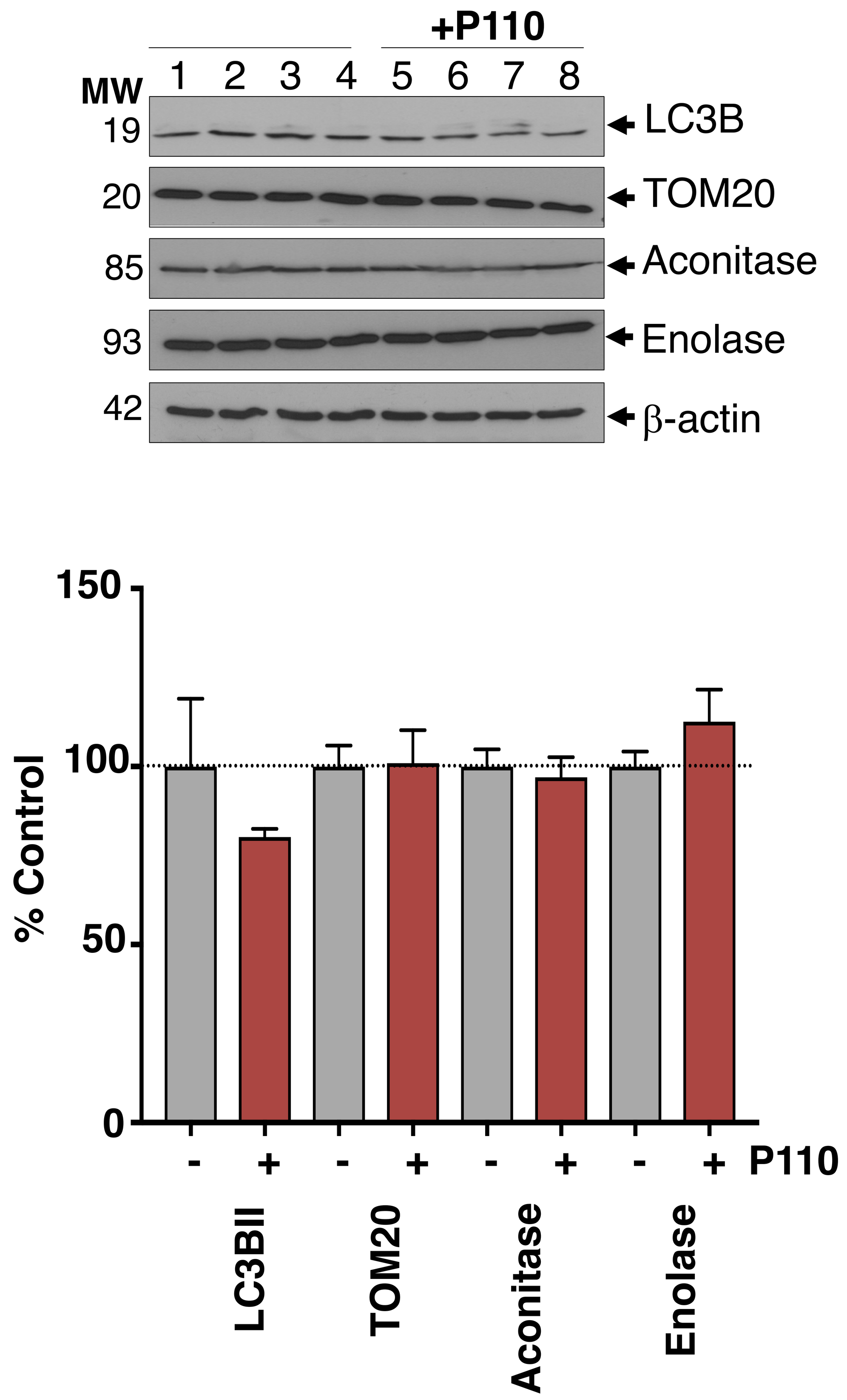

C Cell Stress Markers

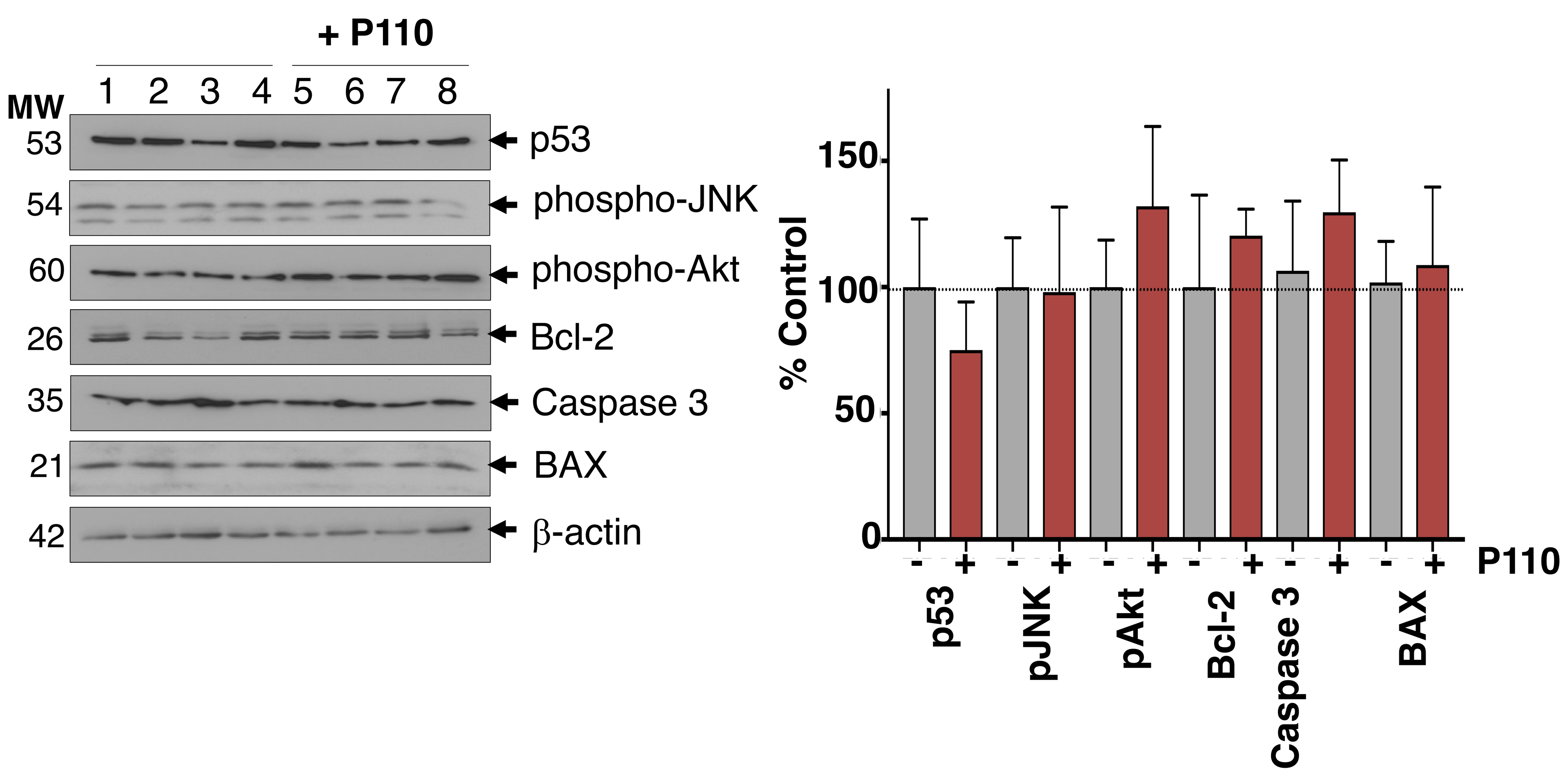

F

	Control		P110				
	Mean	SD	Mean	SD	p-value	unit	n
Glucose	195.3	20.5	205.4	37.5	0.5	mg/dL	10/ group
AST	100.0	47.1	84.3	20.7	0.4	U/L	
ALT	44.1	20.6	41.6	14.9	0.8	U/L	
Alkaline Phosphatase	74.8	27.5	67.3	29.3	0.6	IU/L	
Total Bilirubin	0.2	0.1	0.2	0.1	0.6	mg/dL	
BUN	30.6	9.5	29.6	10.7	0.2	mg/dL	
Creatinine	0.5	0.2	0.4	0.2	0.4	mg/dL	
T.Protein	5.5	0.4	5.5	0.6	0.9	g/dL	
Albumin	3.1	0.2	3.0	0.3	0.8	g/dL	
Globulin	2.5	0.2	2.4	0.3	0.6		
Carbon Dioxide	16.6	2.1	16.4	2.1	0.9	mmol/L	
Na/K Ratio	18.4	3.5	16.3	2.1	0.2		
Anion Gap	31.5	3.2	32.2	2.7	0.7	mmol/L	

	Control		P110				
	Mean	SD	Mean	SD	p-value	unit	n
WBC	12.1	2.5	12.8	1.6	0.6	K/uL	5/ group
RBC	9.1	0.8	9.8	0.7	0.2	M/uL	
Hgb	12.8	1.6	14.0	1.0	0.2	gm/dL	
HCT	42.8	4.3	47.5	3.6	0.1	%	
MCV	47.2	1.3	48.4	1.9	0.3	fL	
MCH	14.1	0.6	14.3	0.3	0.7	pg	
MCHC	32.5	1.8	31.7	1.5	0.5	g/dL	
Platelet count	1086.2	366.2	1048.4	274.9	0.9	K/uL	
RDW	18.6	4.4	19.9	3.2	0.6	%	
Reticulocyte Count	4.7	0.7	4.9	1.0	0.7	%	
IRF	54.3	1.3	56.5	2.8	0.2	%	
Neutrophils	23.0	12.4	17.8	9.3	0.5	%	
Lymphocytes	70.2	13.8	76.0	11.4	0.5	%	
Monocytes	6.0	2.9	4.6	0.9	0.3	%	
Eosinophils	2.0	1.6	1.8	1.3	0.8	%	

Appendix Figure S1.

A. Bright field image of an H&E stained sections showing normal liver architecture, and components of basic liver lobules, with portal area and central venule in both naïve and P110 treated mice. Similarly no changes were observed in the spleen size or morphology after 5-month treatment with vehicle or P110 at 3mg/kg/day in naïve mice ;$n = 10$.

B. Groom and rearing frequency as well as duration were analyzed using activity chamber and had no changes after 5-month treatment with vehicle or P110 at 3mg/kg/day in naïve mice; $n = 10$.

C. Protein levels of cell stress markers including p53, pJNK, pAkt, Bcl-2, Caspase 3 and BAX were measured after 5-month treatment with vehicle or P110 at 3mg/kg/day in naïve mice showed no significant alterations. β-actin was used as a loading control. Protein levels were quantified and represented as % control; $n = 4$.

D. Protein levels of mitochondrial fission and fusion proteins, including Drp1, Fis1, Mff, Mid49, Mid51, Mfn1, Mfn2 and Opa1 were measured after 5-month treatment with vehicle or P110 at 3mg/kg/day in naïve mice and showed no significant alterations. β-actin was used as a loading control. Protein levels were quantified and represented as % control; $n = 4$.

E. Protein levels of mitochondrial heath markers, including LC3BII, TOM20, Aconitase and Enolase were measured after 5-month treatment with vehicle or P110 at 3mg/kg/day in naïve mice and showed no significant alterations. β-actin was used as a loading control. Protein levels were quantified and represented as % control; $n = 4$.

F. Hematology ($n = 5$) & Clinical Chemistry ($n = 5$) Testing was performed after 5-month treatment with vehicle or P110 at 3mg/kg/day in naïve mice and showed no significant alterations.

Data information: An experimenter who was blind to genotypes and drug groups conducted all the behavior and survival studies. Mean, standard deviation, and P-values are shown; probability by one-way ANOVA (with uncorrected Fisher's LSD post hoc test).

Appendix Table 1

List of antibodies used for western blot analysis:

Antibody name	Company	Catalog No	Dilution
Anti-Aconitase	Abcam	126595	1:1000
Anti-ATF6	Novus Biologicals	NBP1-40256	1:500
Anti-BAX (Active)	Enzo Life Sciences	ALX-804-224-C100	1:500
Anti-Bcl-2	Santa Cruz Biotechnology	sc-492	1:500
Anti-CHOP	Cell Signaling Technology	2895	1:500
Anti-cytochrome c	BD Pharmingen™	556432	1:2000
Anti-Drp1	BD Transduction Laboratories™	611113	1:500
Anti-eIF2a	Cell Signaling Technology	9722	1:200
Anti-Enolase	Santa Cruz Biotechnology	sc-15343	1:1000
Anti-Fis1	Proteintech	10956-1-AP	1:1000
Anti-GRP78	Abcam	21685	1:500
Anti-JNK	Cell Signaling Technology	9252	1:500
Anti-LC3BII	Cell Signaling Technology	3868	1:500
Anti-Mff	Proteintech	17090-1-AP	1:500
Anti-Mfn1	Proteintech	13798-1-AP	1:500
Anti-Mfn2	Proteintech	12186-1-AP	1:500
Anti-Mid49	Proteintech	16413-1-AP	1:500
Anti-Mid51	Proteintech	20164-1-AP	1:500
Anti-Opa1	Santa Cruz Biotechnology	sc-393296	1:500
Anti-p53	Cell Signaling Technology	9282	1:1000
Anti-p62	Abcam	56416	1:500
Anti-Parkin	Abcam	77924	1:500
Anti-phospho-Drp1 (Ser616)	Cell Signaling Technology	3455	1:200
Anti-phospho-Drp1 (Ser637)	Cell Signaling Technology	4867	1:200
Anti-phospho-eIF2a	Cell Signaling Technology	9721	1:500
Anti-phospho-JNK	Cell Signaling Technology	9251	1:200
Anti-TOM20	Santa Cruz Biotechnology	sc-11415	1:1000
Anti-VDAC1	Abcam	14734	1:2000
Anti-XBP1	Abcam	37152	1:500
Anti-β-actin	Cell Signaling Technology	3700	1:1000
