# Supplementary material for: Inhibition of Drp1/Fis1 interaction slows progression of amyotrophic lateral sclerosis
Source: EMBO Mol Med. 2018 Jan 15;10(3):e8166. doi: 10.15252/emmm.201708166 (PMC5840540; doi:10.15252/emmm.201708166)
Supplement: Supplementary file 2 — Expanded View Figures PDF [file EMMM-10-e8166-s002.pdf]

Expanded View Figures

Figure EV1. Mitochondrial dysfunction and autophagy activation in ALS patient-derived fibroblasts.

A Representative Western blots showing siRNA-mediated reduction in total Fis1 levels. *n* = 3.
B Mitochondrial elongation in healthy control fibroblasts and ALS patient-derived fibroblasts from the stained TOM20 images was quantified using a macro in ImageJ after Fis1 siRNA treatment. *n* = 3 performed in duplicate. At least 100 cells/group were analyzed while blinded to experimental conditions.
C Mitochondrial interconnectivity in healthy control fibroblasts and ALS patient-derived fibroblasts from the stained TOM20 images was quantified using a macro in ImageJ after Fis1 siRNA; *n* = 3 performed in duplicate. At least 100 cells/group were analyzed while blinded to experimental conditions.
D Representative Western blots showing Drp1 and p62 association with mitochondrial-enriched fractions in different ALS patient-derived fibroblasts following culturing in the presence or absence of P110 (1 μM for 24 h); VDAC was used as a loading control for mitochondrial fraction; *n* = 3.
E Levels of p62 in mitochondrial fractions were measured by immunoblotting; VDAC was used as a loading control. Protein levels were quantified and presented as fold change of control 1. Results are presented as fold of control (means ± SD). *n* = 3 performed in triplicate; probability by one-way ANOVA (with Tukey's *post hoc* test).
F Representative Western blots showing Drp1 and Fis1 total levels in different ALS patient-derived fibroblasts following culturing in the presence or absence of P110 (1 μM for 24 h); β-actin was used as a loading control; *n* = 3.
G Drp1 association with Fis1 was examined by Western blot following co-immunoprecipitation of pooled total lysates from three independent experiments from different ALS patient-derived fibroblasts following treatment in the presence or absence of P110 (1 μM for 24 h). *n* = 3.

Data information: Mean, standard deviation, and *P*-values are shown.
Source data are available online for this figure.

© 2018 The Authors

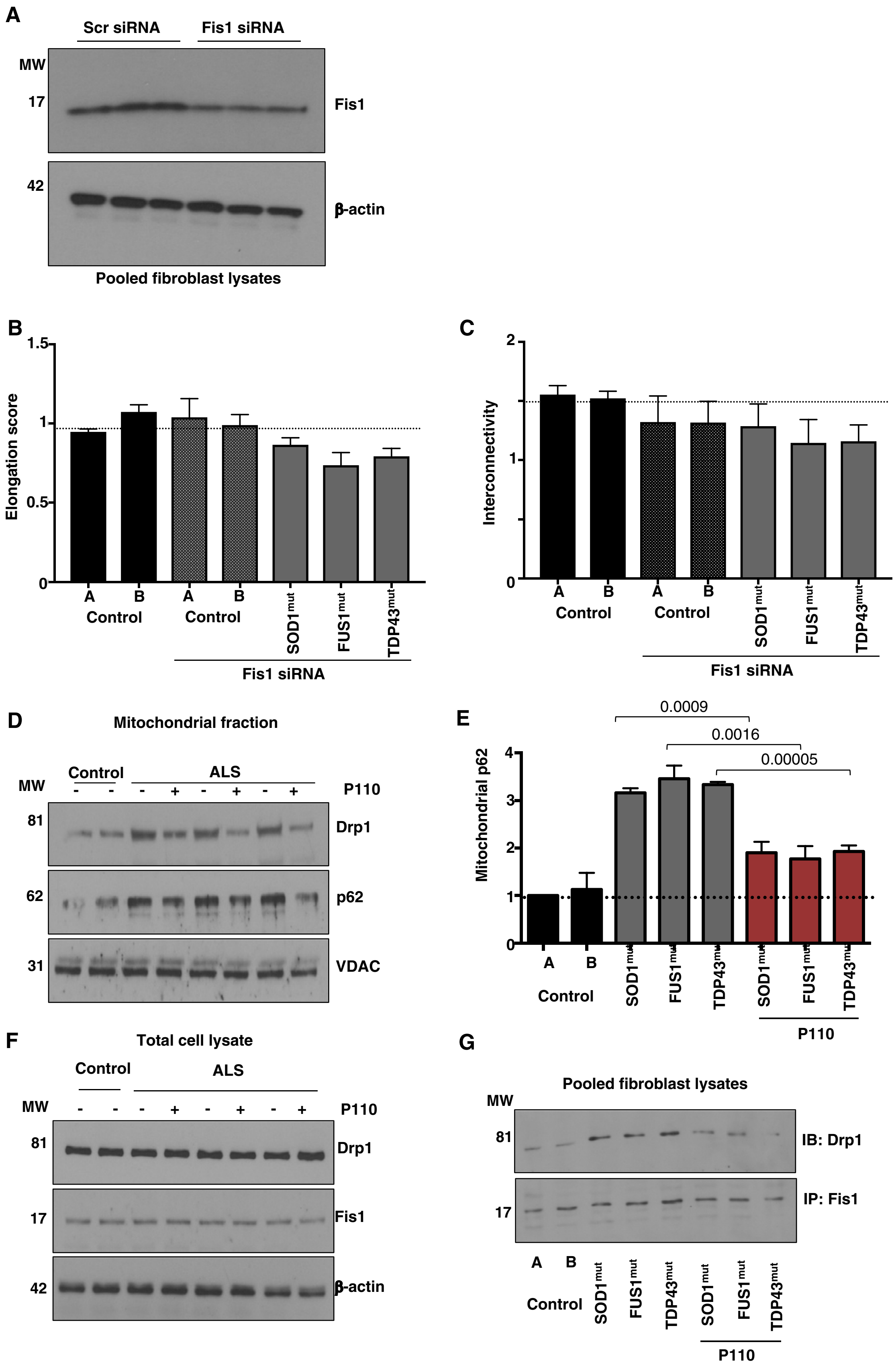
A
Scr siRNA
Fis1 siRNA
MW
17
Fis1
42
β-actin
Pooled fibroblast lysates
B
Elongation score
1.5
1
0.5
0
A B A B
Control Control
SOD1mut FUS1mut TDP43mut
Fis1 siRNA
C
Interconnectivity
2
1
0
A B A B
Control Control
SOD1mut FUS1mut TDP43mut
Fis1 siRNA
D
Mitochondrial fraction
Control
ALS
MW
P110
81
Drp1
62
p62
31
VDAC
E
Mitochondrial p62
0.0009
0.0016
0.00005
A B
Control
SOD1mut FUS1mut TDP43mut SOD1mut FUS1mut TDP43mut
P110
F
Total cell lysate
Control
ALS
MW
P110
81
Drp1
17
Fis1
42
β-actin
G
Pooled fibroblast lysates
MW
81
IB: Drp1
17
IP: Fis1
A B
Control
SOD1mut FUS1mut TDP43mut SOD1mut FUS1mut TDP43mut
P110

Figure EV1.

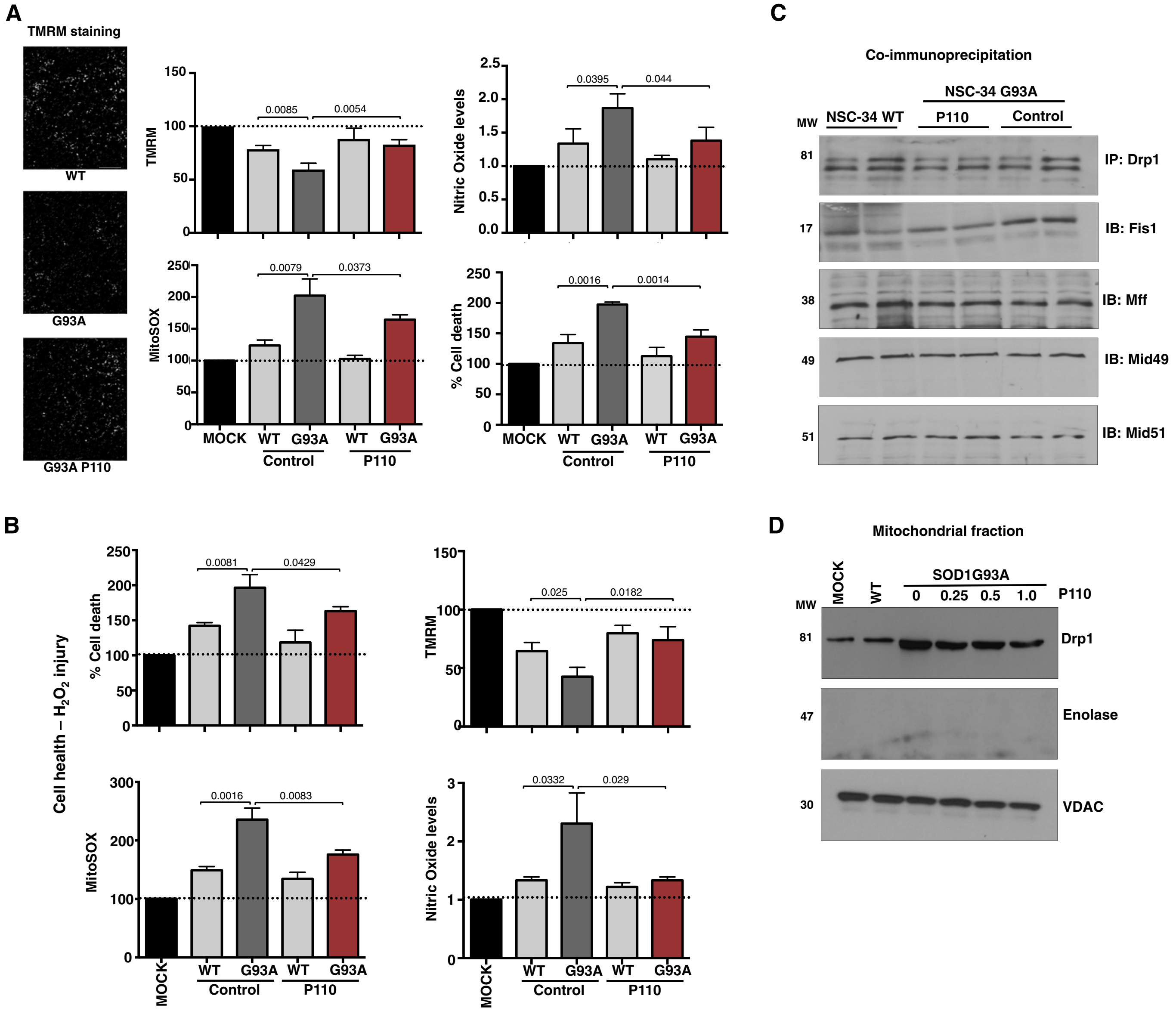

Figure EV2. Mitochondrial dysfunction in hSOD1-WT- and hSOD1-G93A-expressing NSC-34 differentiated cells.

A Mitochondrial health using mitochondrial membrane potential (TMRM) and mitochondria-specific ROS indicator (MitoSOX), nitric oxide levels (Griess reagent), and cell death (using LDH assay) in hSOD1-WT- and hSOD1-G93A-expressing NSC-34 differentiated cells cultured in the presence or absence of P110 (1 μM for 24 h). Results are presented as percent or fold of MOCK (empty vector); *n* = 3 performed in quintuplet; probability by one-way ANOVA (with Tukey's *post hoc* test). Scale bar: 50 μm.

B Cell death (using LDH assay), mitochondrial-specific ROS production (MitoSOX), mitochondrial membrane potential (using TMRM), and nitric oxide levels in hSOD1-WT- and hSOD1-G93A-expressing NSC-34 differentiated cells were determined following H_2O_2 injury in the presence or absence of P110 (1 μM/24 h); *n* = 3 performed in quintuplet; probability by one-way ANOVA (with Tukey's *post hoc* test).

C Co-immunoprecipitation of Drp1 with Fis1, Mff, Mid49, or Mid51 was analyzed in hSOD1-WT- and hSOD1-G93A-expressing NSC-34 differentiated cells, cultured in the presence or absence of P110 (1 μM for 24 h). *n* = 2 performed in duplicates (pooled).

D Levels of Drp1 were determined in mitochondrial fractions by immunoblotting in hSOD1-WT- and hSOD1-G93A-expressing NSC-34 cells as above, cultured in the presence or absence of P110 (0.25, 0.5, 1 μM for 24 h); VDAC, a mitochondrial membrane protein, was used as a loading control, whereas enolase, a cytosolic marker, was used to assess the mitochondrial sample purity. *n* = 2 performed in duplicates (pooled).

Data information: Mean, standard deviation, and *P*-values are shown. Results are presented as percent or fold of MOCK (empty vector).
Source data are available online for this figure.

 © 2018 The Authors

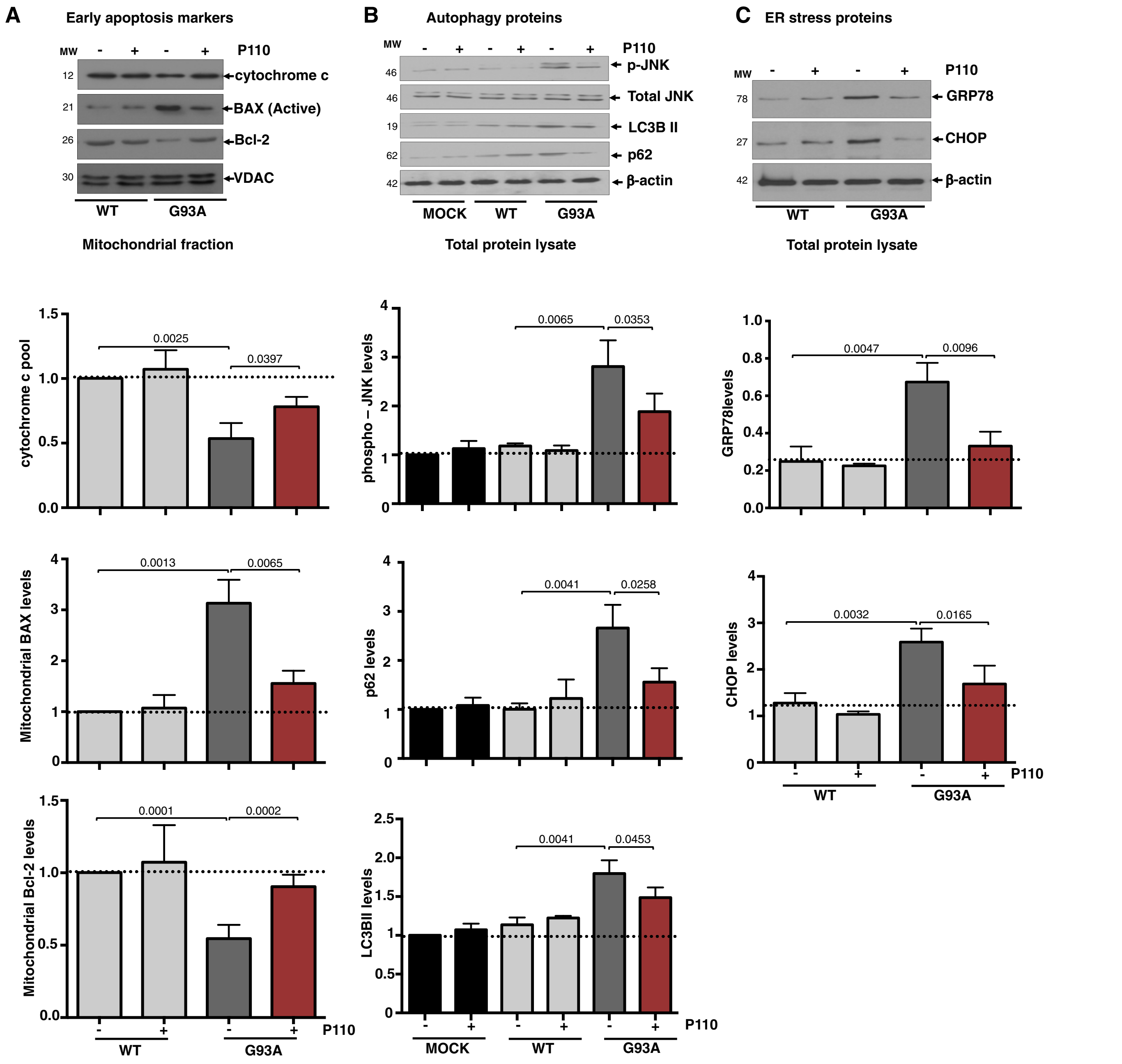

Figure EV3. Increases apoptotic markers in hSOD1-G93A-expressing NSC-34 differentiated cells relative to hSOD1-WT.

A Representative Western blots showing levels of cytochrome c, BAX, and Bcl-2 were examined in mitochondrial fractions by immunoblotting in hSOD1-WT- and hSOD1-G93A-expressing NSC-34 differentiated cells under serum starvation in the presence or absence of P110 (1 μM/24 h); VDAC. Protein levels were quantified and presented as fold change of hSOD-1 WT.

B Representative Western blots showing levels of autophagy-associated proteins; Parkin and LC3BII examined in mitochondrial fractions. Representative Western blots showing phosphorylated-JNK, total JNK, p62, and LC3BII total levels in hSOD1-WT- and hSOD1-G93A-expressing NSC-34 differentiated cells under serum starvation in the presence or absence of P110 (1 μM/24 h); VDAC and β-actin were used as a loading control for mitochondrial fraction (top panels) and total fraction (lower panels), respectively. Protein levels were quantified and presented as fold change of hSOD-1 WT.

C Representative Western blots showing levels of ER stress-associated protein, GRP78, CHOP, in hSOD1-WT- and hSOD1-G93A-expressing NSC-34 differentiated cells under serum starvation in the presence or absence of P110 (1 μM/24 h); β-actin was used as a loading control. Protein levels were quantified and presented as fold change of hSOD-1 WT.

Data information: Mean, standard deviation, and *P*-values are shown. *n* = 3 performed in (A) duplicates or (B, C) quintuplet; probability by one-way ANOVA (with Tukey's *post hoc* test).
Source data are available online for this figure.

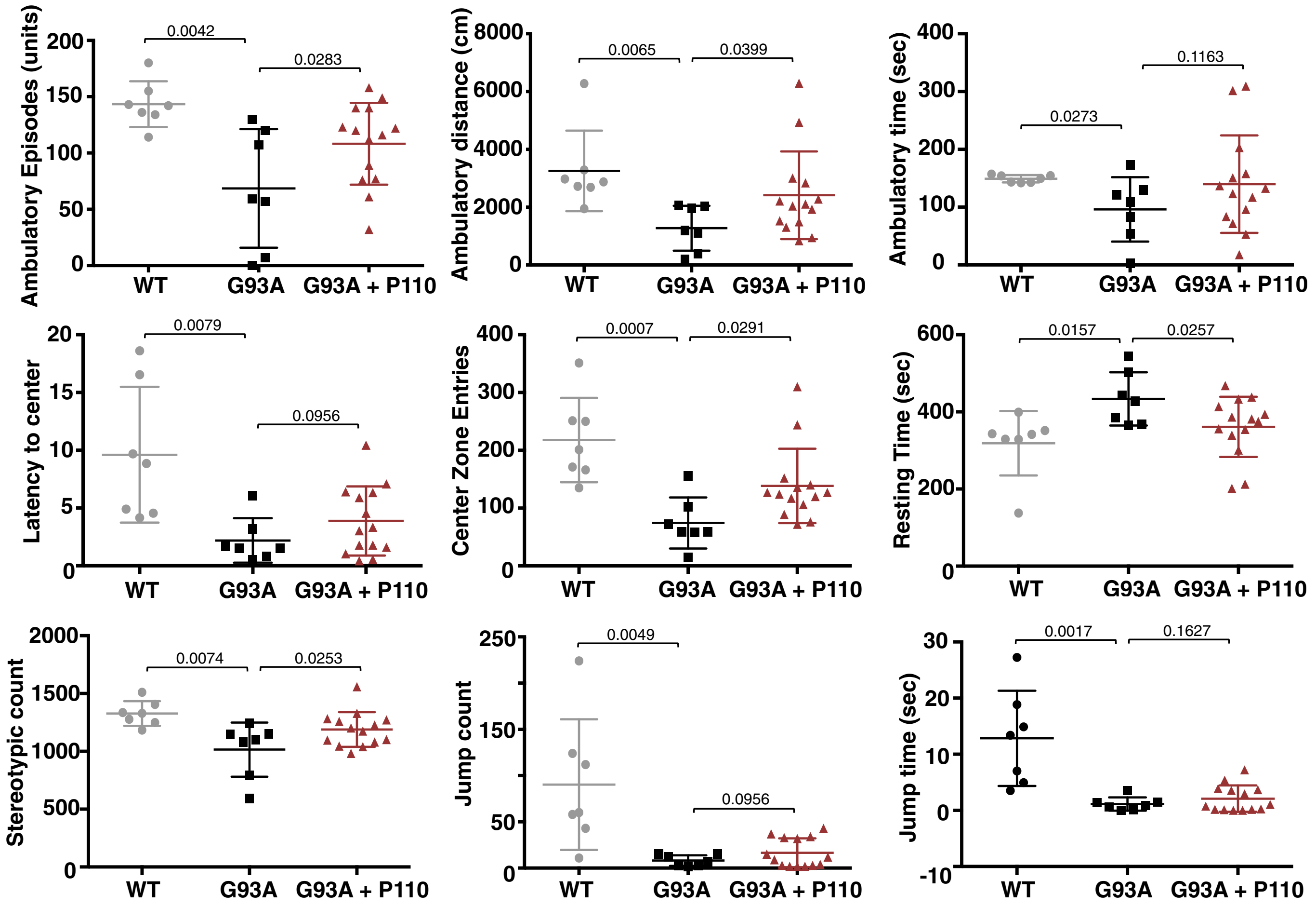

Figure EV4. Inhibition of Drp1 association with Fis1 using P110 in the symptomatic phase of slows disease progression in SOD1 ALS mouse model.

Ambulatory distance, time, episodes, resting time, latency toward center of the chamber, number of center zone entries, stereotypic counts, jump counts, and jump times were analyzed using activity chamber after 24 days of treatment with vehicle or P110 at 3 mg/kg/day in G93A SOD1 mice. *n* = 7 for WT mice; *n* = 7 for G93A ALS mice + TAT; *n* = 14 for G93A ALS mice + P110; probability by one-way ANOVA (with uncorrected Fisher's LSD *post hoc* test). An experimenter who was blind to genotypes and drug groups conducted all the behavior and survival studies. Mean, standard deviation, and *P*-values are shown.

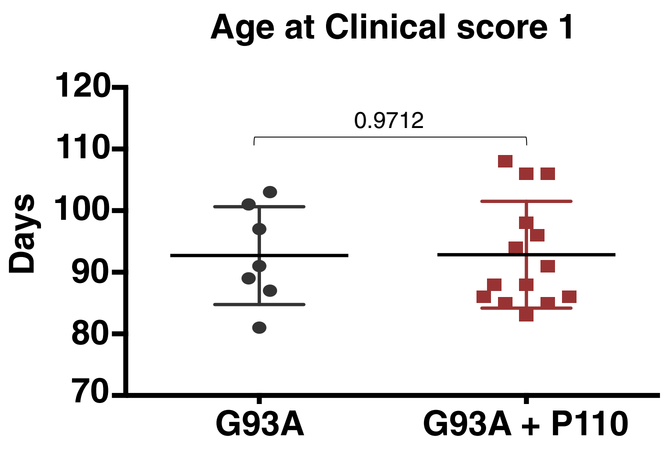

Figure EV5. No difference in the age of disease onset.

There was no significant difference in the onset of clinical score 1 between the two groups. *n* = 7 for G93A ALS mice + TAT; *n* = 14 for G93A ALS mice + P110; probability by one-way ANOVA (with uncorrected Fisher's LSD *post hoc* test). An experimenter who was blind to genotypes and drug groups conducted all the behavior and survival studies. Mean, standard deviation, and *P*-values are shown.

© 2018 The Authors
