# Supplementary figures and images for: Inhibition of Drp1/Fis1 interaction slows progression of amyotrophic lateral sclerosis
Source: EMBO Mol Med. 2018 Jan 15;10(3):e8166. doi: 10.15252/emmm.201708166 (PMC5840540; doi:10.15252/emmm.201708166)

Source Data Fig EV1G.

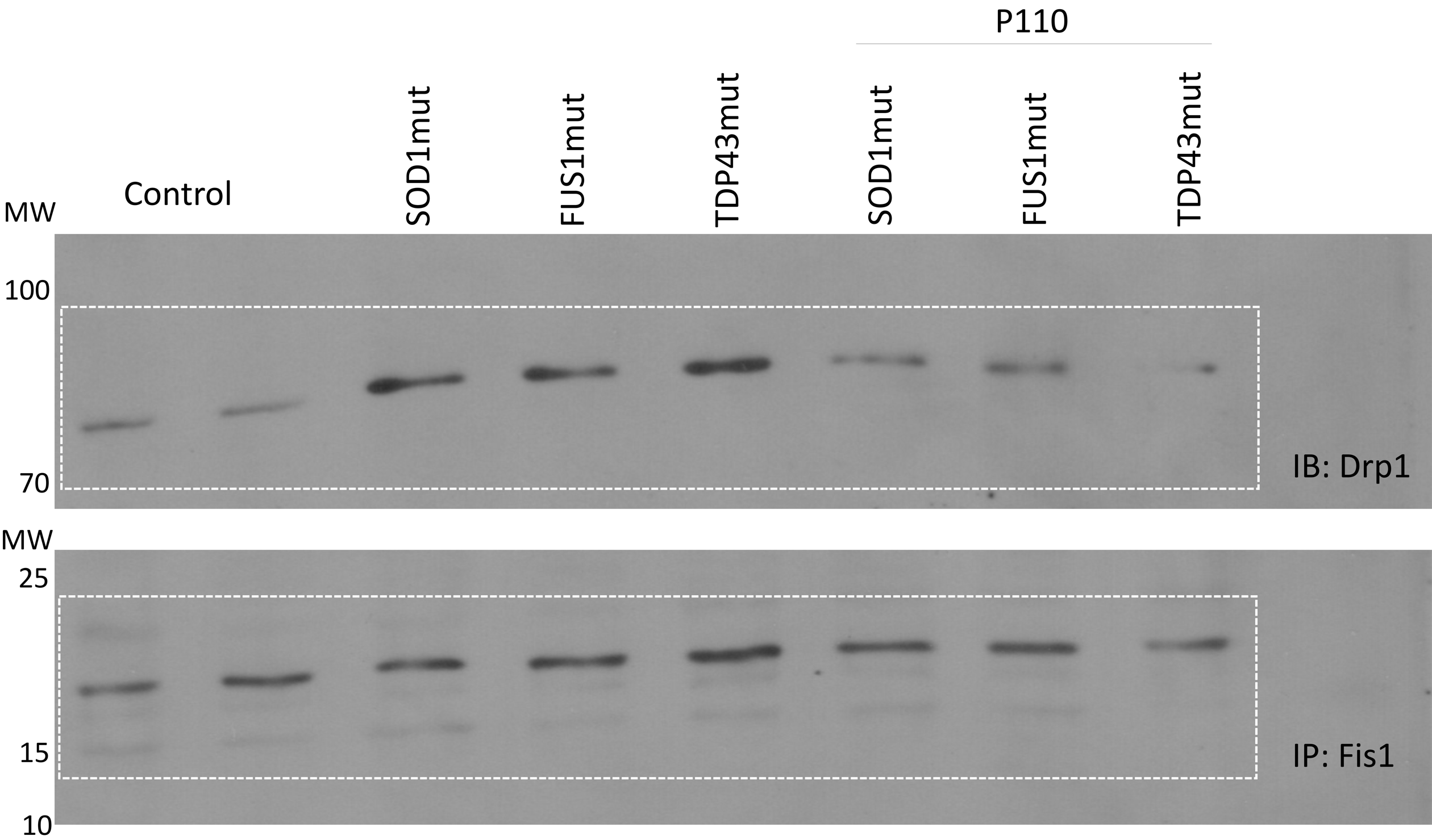

Supplement: Supplementary file 3 — Source Data for Expanded View [file EMMM-10-e8166-s005.zip › Source_data_EV1G.pdf]

Source Data Fig EV2C.

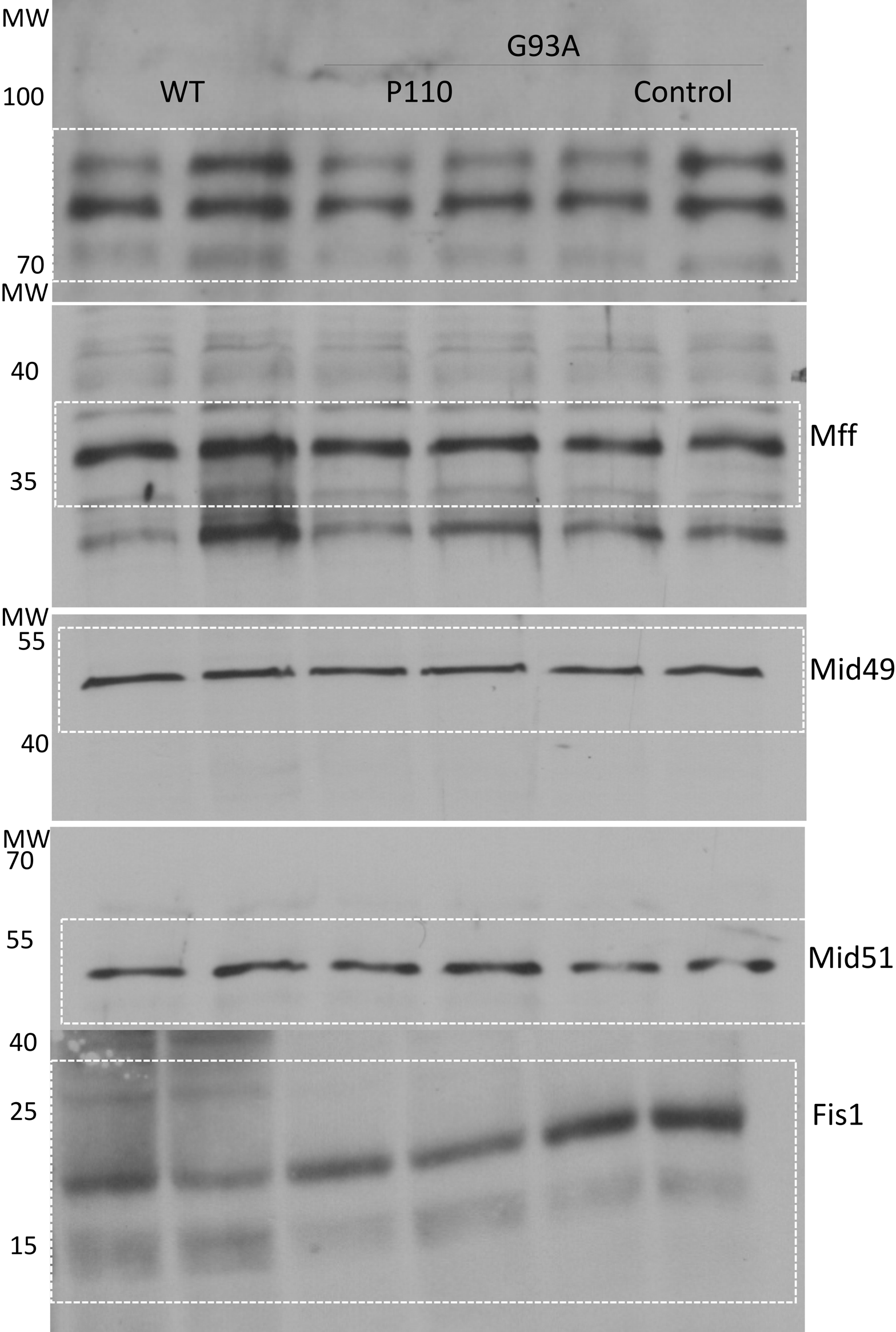

Supplement: Supplementary file 3 — Source Data for Expanded View [file EMMM-10-e8166-s005.zip › Source_data_EV2C.pdf]

Source Data Fig EV3B.

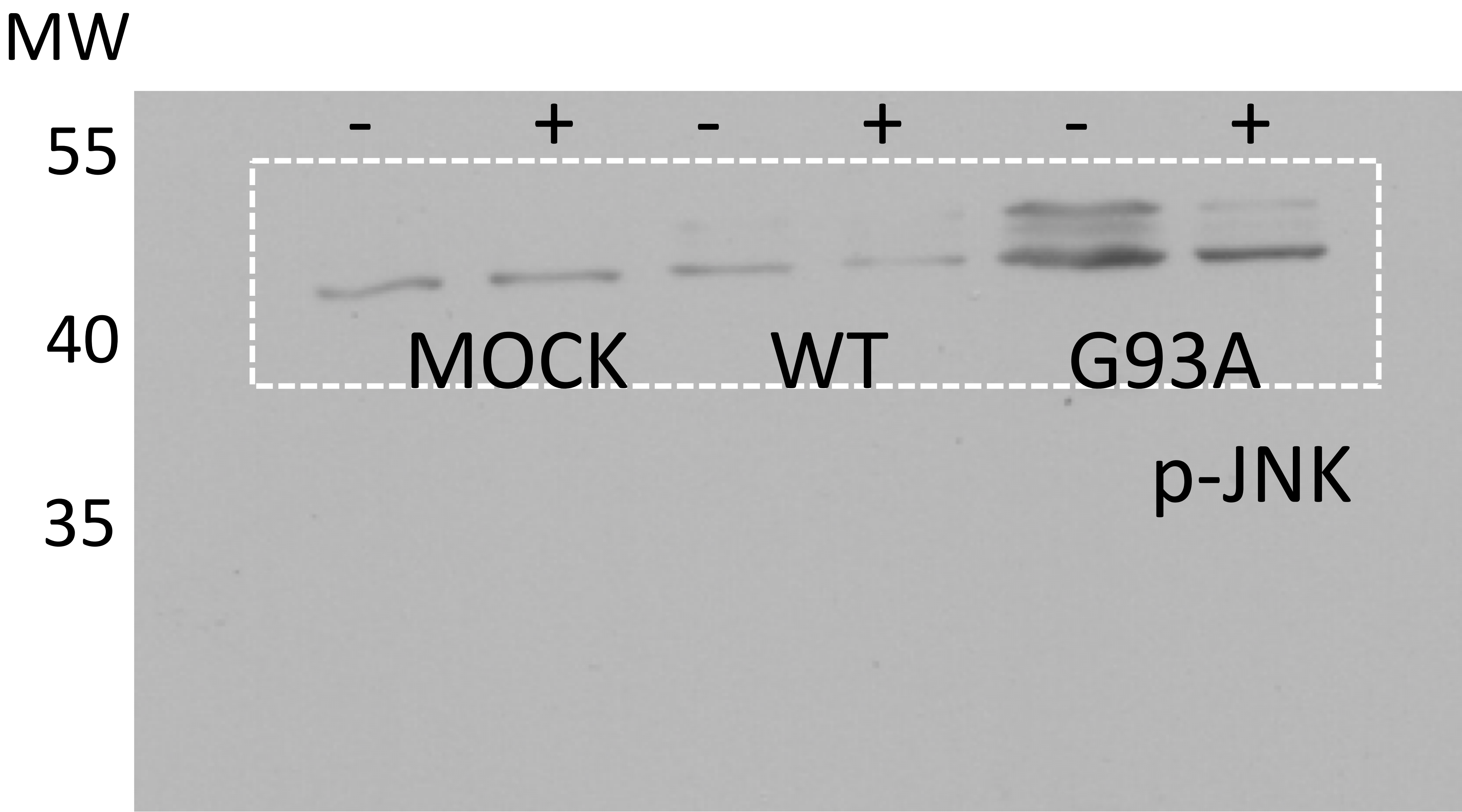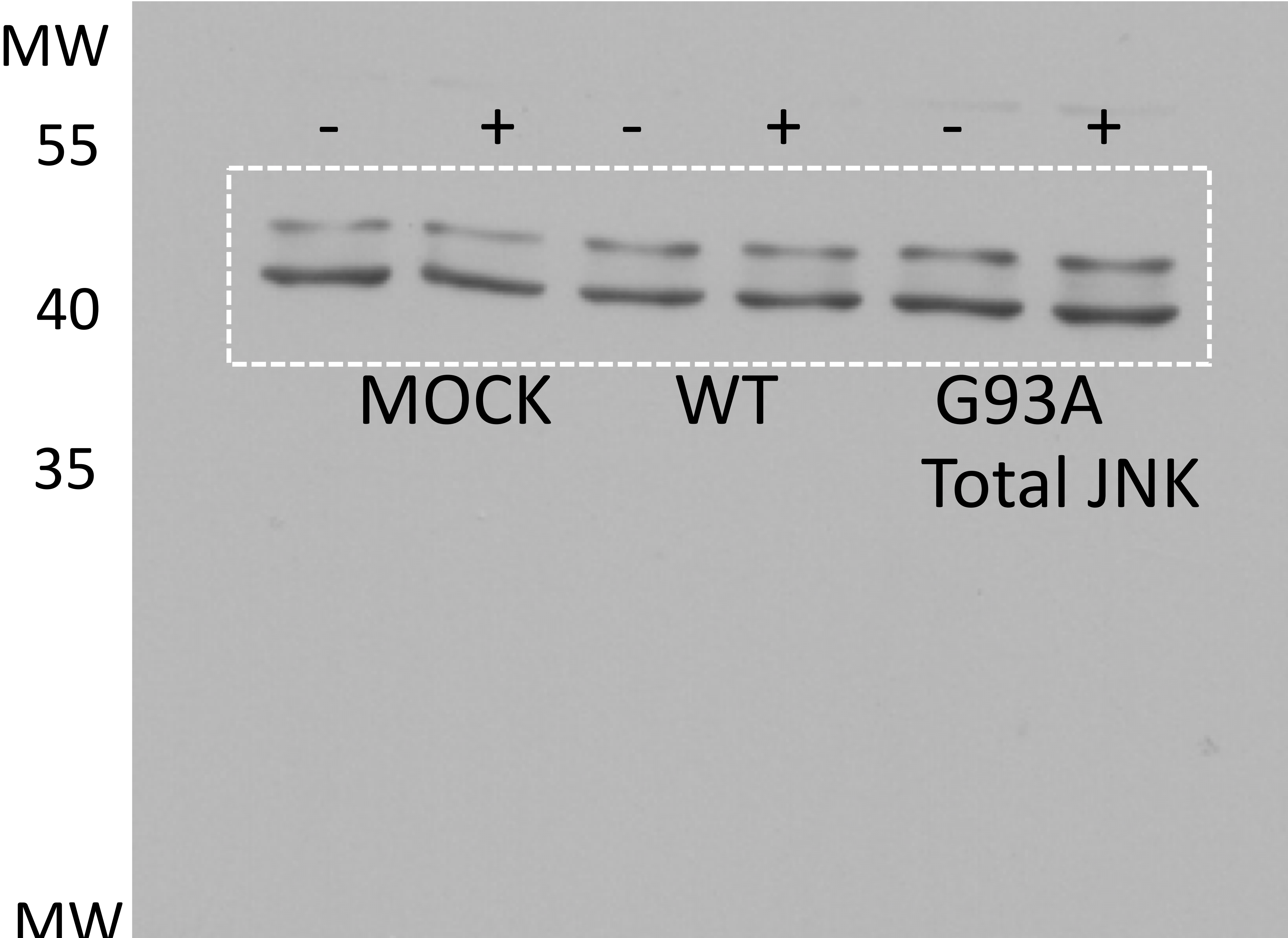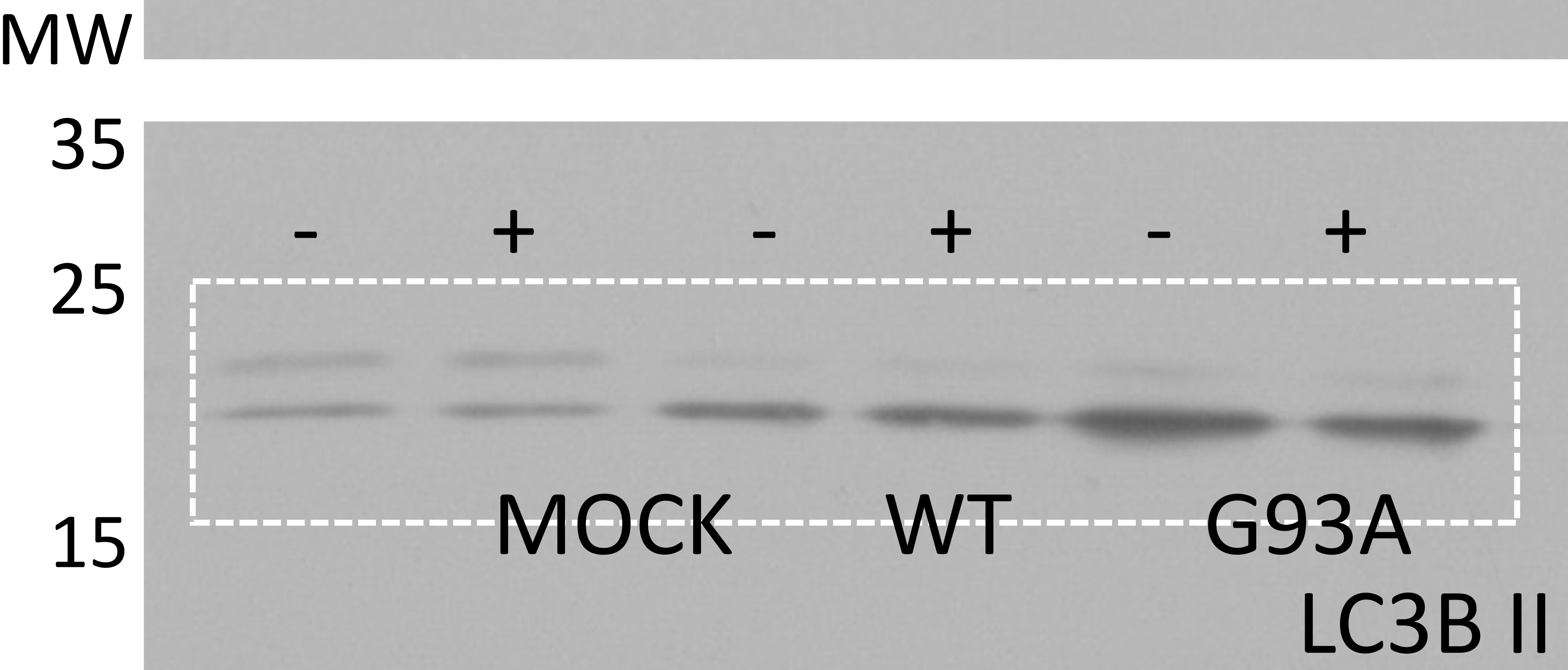

Supplement: Supplementary file 3 — Source Data for Expanded View [file EMMM-10-e8166-s005.zip › Source_data_EV3B.pdf]

Source Data Fig 2C.

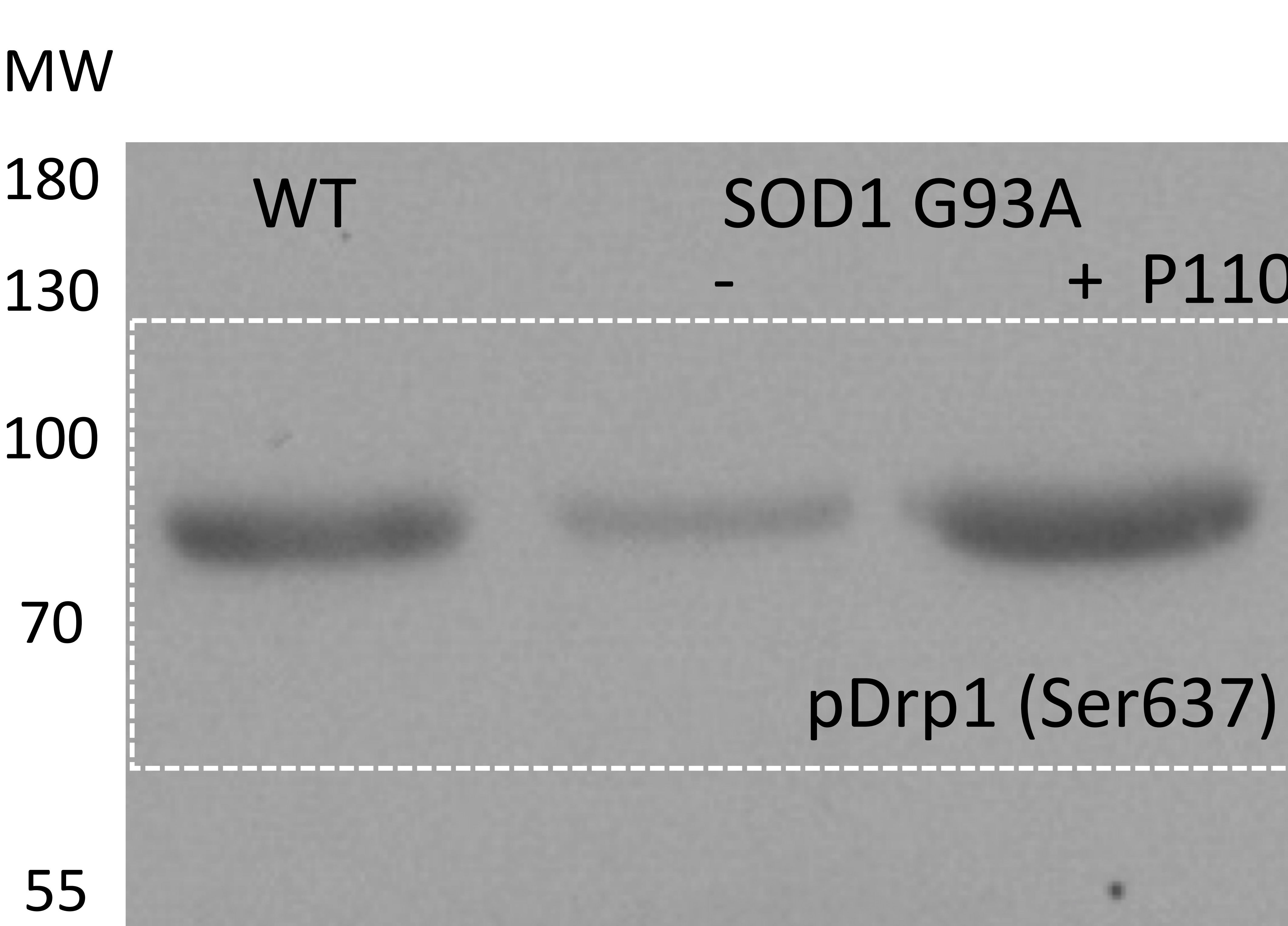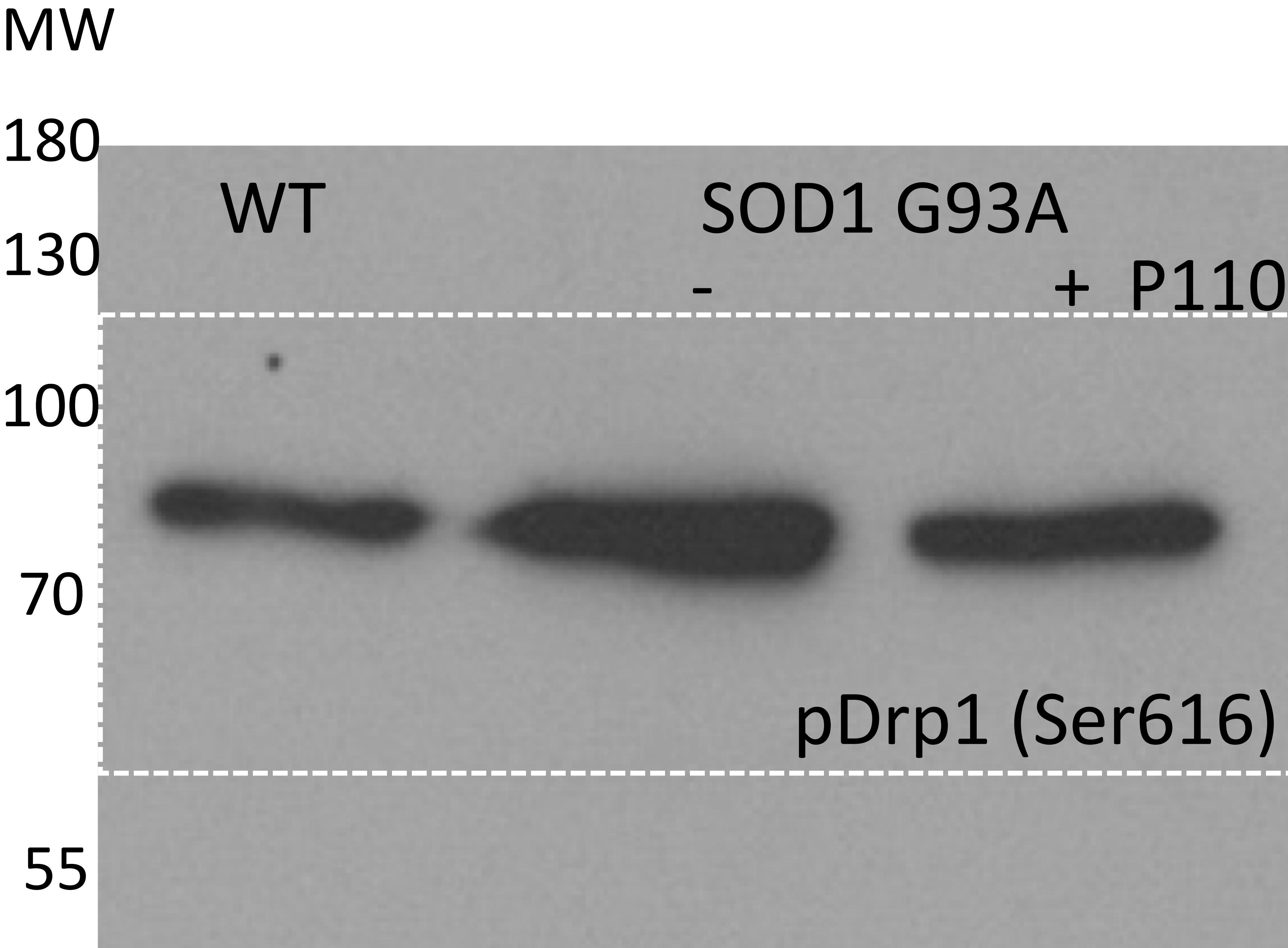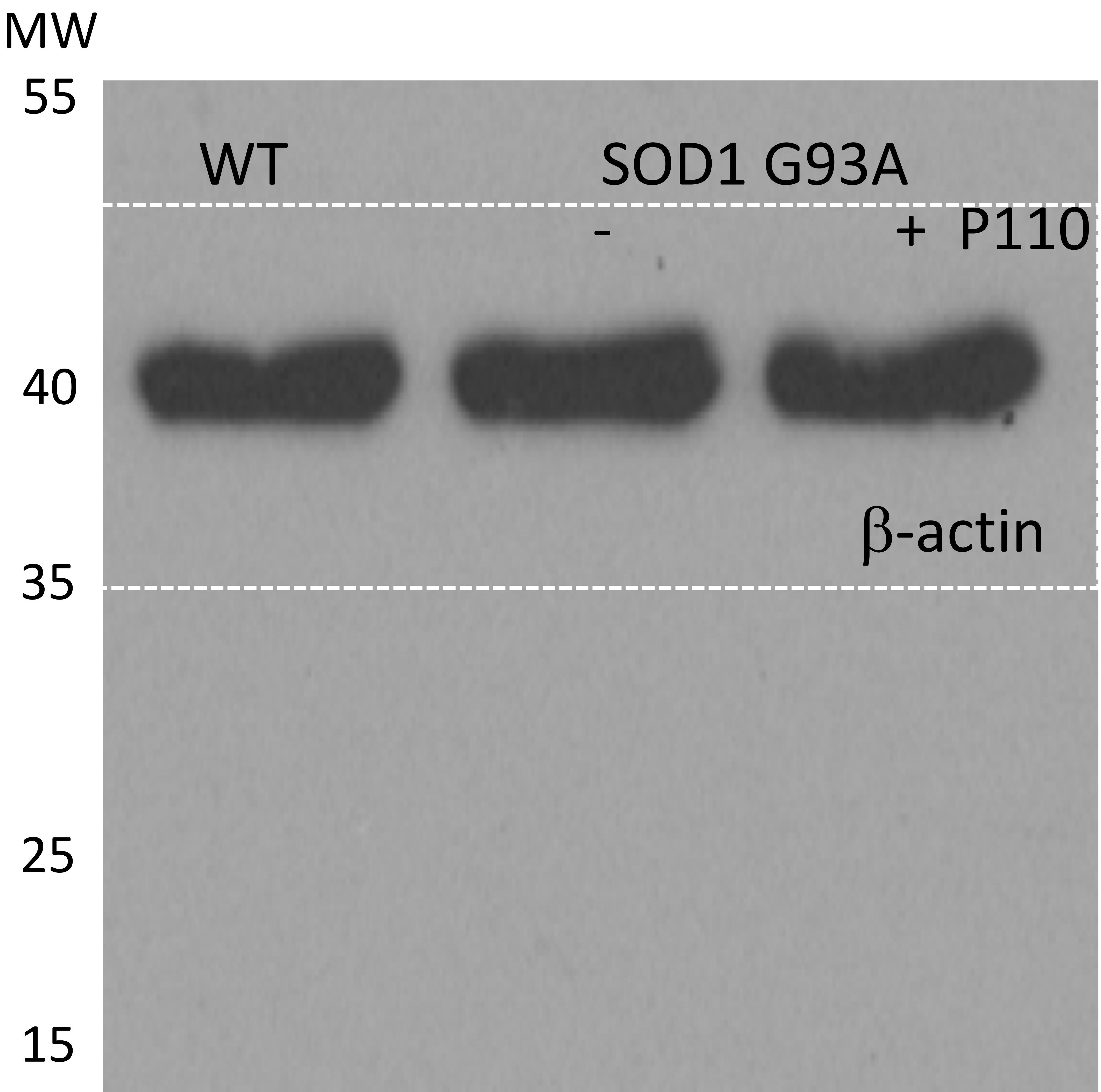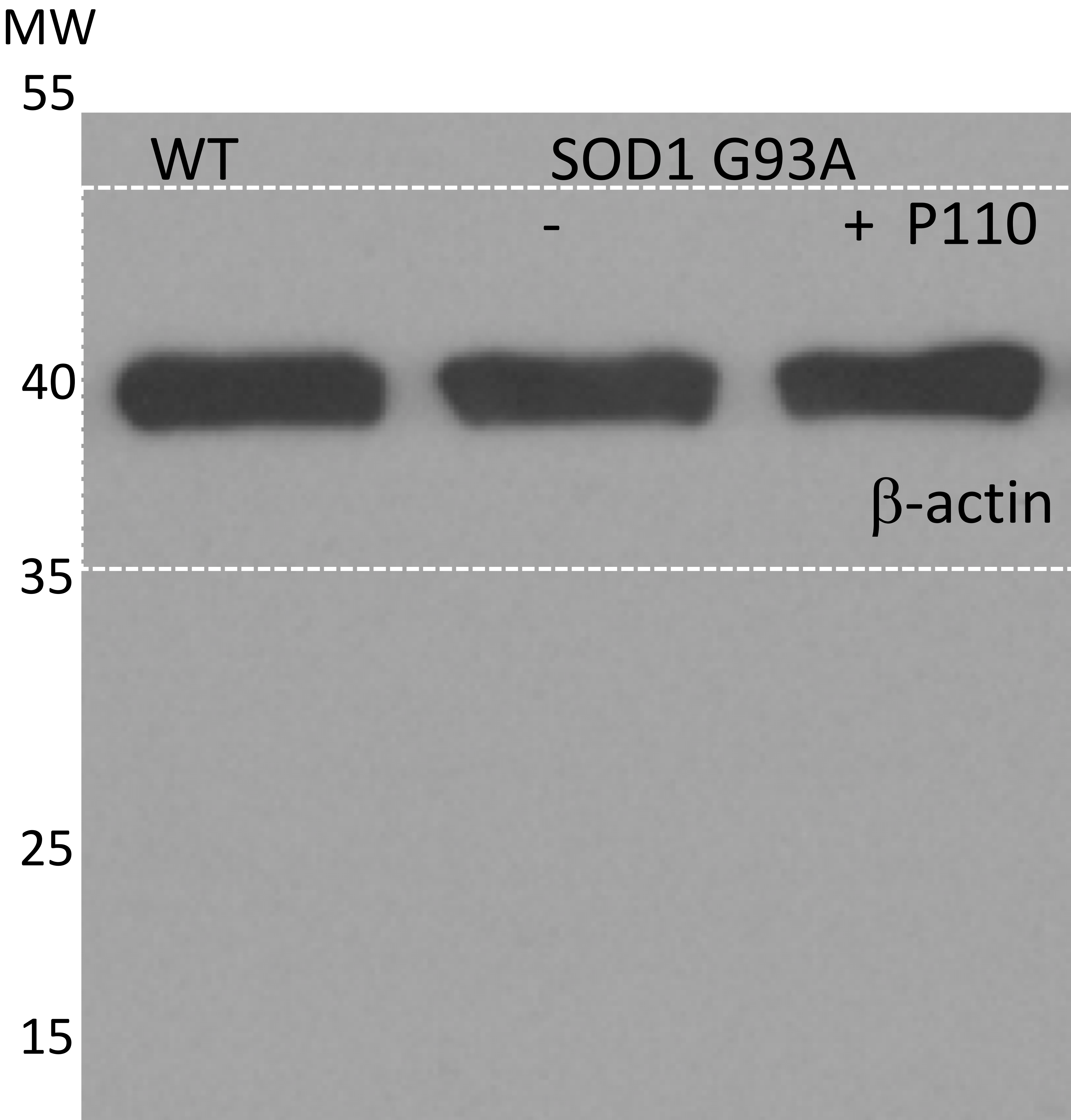

Supplement: Supplementary file 5 — Source Data for Figure 2 [file EMMM-10-e8166-s003.pdf]

Source Data Fig 5G.

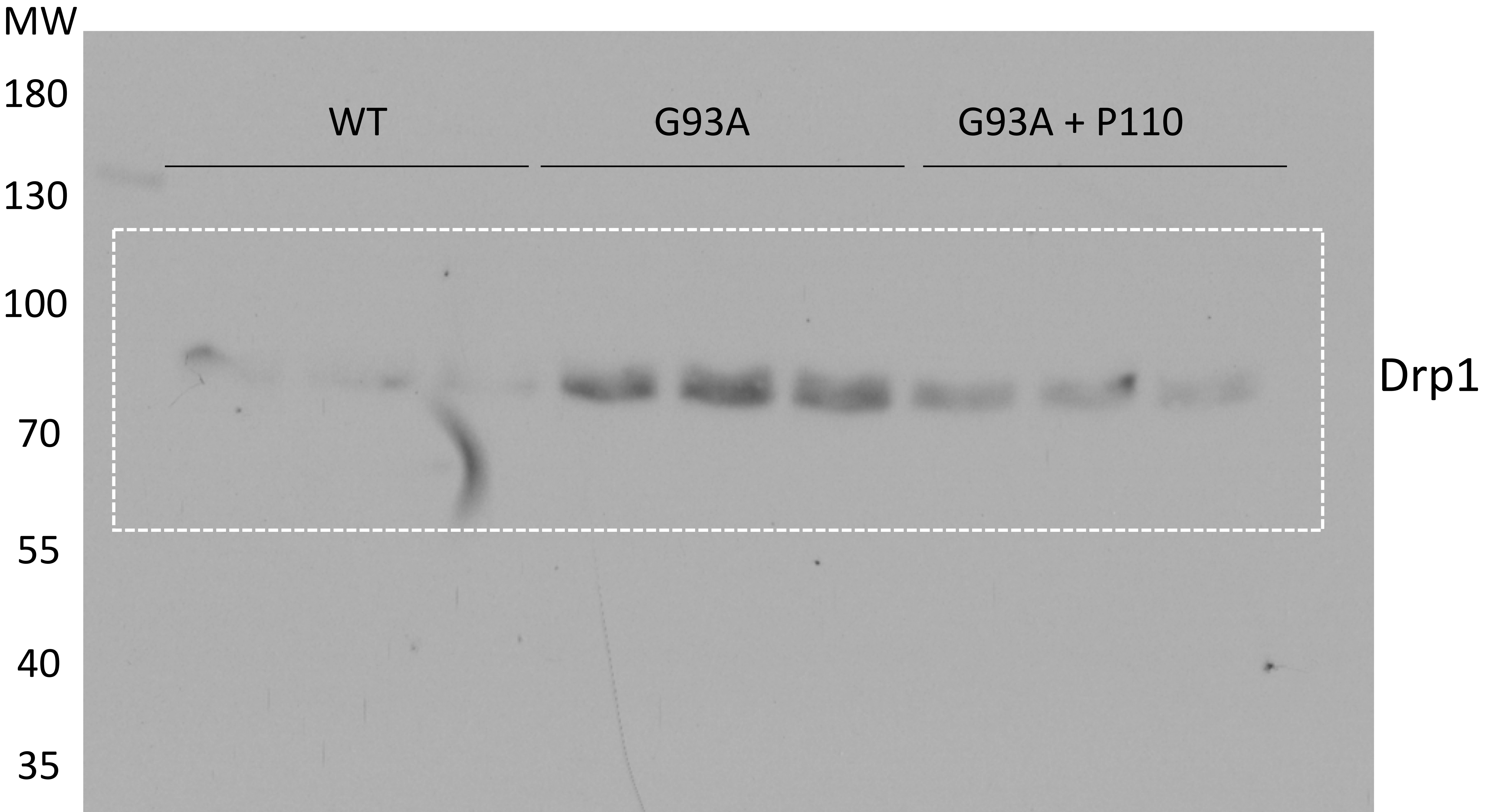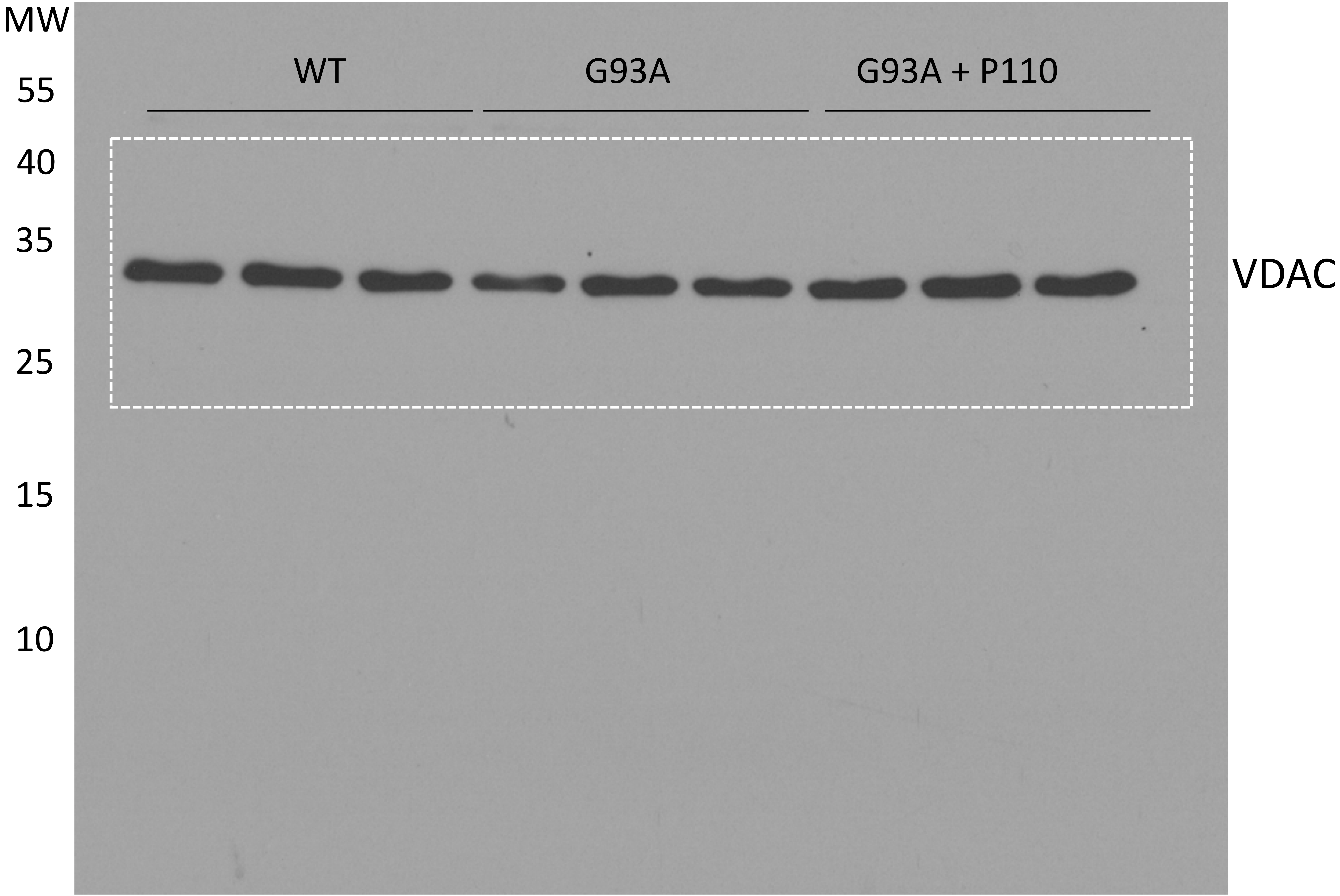

Supplement: Supplementary file 6 — Source Data for Figure 5 [file EMMM-10-e8166-s004.pdf]
